# War-Time Work in an Infirmary

**Published:** 1918-05-04

**Authors:** 


### War-time Work in an Infirmary.

Dr. Lionel Baly, medical superintendent of the Lambeth Infirmary, in his annual report on the past year's work in the infirmary, is of opinion that the slight decrease in the number of cases has been more than made up by the acute character of the work. The increase in the number of beds available had not relieved the pressure on the wards, because no fewer than five wards were given up entirely to cases of whooping-cough and measles. That the work approximated to that of a general hospital was shown by the number of accident cases. No fewer than sixty-six were admitted for fractured femur. Dr. Baly says that it is becoming more difficult to obtain fit and competent men to perform the duties of male attendants, receiving wardsmen, etc. From the commencement of the war only five exemptions have been asked for and granted by the Tribunal up to the present time. It was doubtful if any efficient substitutes would be obtainable for some of the male staff who would come under the new Military Service Act raising the age to fifty-one years. Should this prove to be the case, and the present exemptions be cancelled, the result was likely to be serious, because other institutions were not performing the work usually expected of them. He had thought it necessary to bring to the notice of the Local Government Board a few clear cases showing that the civil population, especially children, were beginning to suffer seriously for want of medical treatment. He was not aware that any other institution was prepared to undertake the work performed at the infirmary. We have all suffered so much from panic legislation that a breakdown in the Medical Service is likely to be the only argument to gain a hearing.

